# Sudden Fall Detection of Human Body Using Transformer Model

**DOI:** 10.3390/s24248051

**Published:** 2024-12-17

**Authors:** Duncan Kibet, Min Seop So, Hahyeon Kang, Yongsu Han, Jong-Ho Shin

**Affiliations:** Department of Industrial Engineering, Chosun University, Gwangju 61452, Republic of Korea; duncankibet90@gmail.com (D.K.); thalstjq01@naver.com (M.S.S.); gkgus0113@gmail.com (H.K.); hanys6202@naver.com (Y.H.)

**Keywords:** fall detection, transformers, speed-based anomaly detection, pose estimation, time-series analysis

## Abstract

In human activity recognition, accurate and timely fall detection is essential in healthcare, particularly for monitoring the elderly, where quick responses can prevent severe consequences. This study presents a new fall detection model built on a transformer architecture, which focuses on the movement speeds of key body points tracked using the MediaPipe library. By continuously monitoring these key points in video data, the model calculates real-time speed changes that signal potential falls. The transformer’s attention mechanism enables it to catch even slight shifts in movement, achieving an accuracy of 97.6% while significantly reducing false alarms compared to traditional methods. This approach has practical applications in settings like elderly care facilities and home monitoring systems, where reliable fall detection can support faster intervention. By homing in on the dynamics of movement, this model improves both accuracy and reliability, making it suitable for various real-world situations. Overall, it offers a promising solution for enhancing safety and care for vulnerable populations in diverse environments.

## 1. Introduction

As many countries transition into aging societies, the number of elderly people is increasing, many of whom live in one- or two-member households. In such family compositions, emergency situations such as sudden falls or heart attacks are particularly dangerous since it is difficult to receive immediate assistance from other family members. To address this issue, various technologies to monitor and assist elderly people have been developed. Activities of Daily Living (ADLs) are essential for assessments in fall detection studies, especially those involving balance. The Berg Balance Scale (BBS), commonly used for evaluating balance among older adults, includes tasks representative of daily life scenarios where falls may occur [1]. Among them, sudden fall detection is regarded as one of the most urgent technologies. Recent advancements in artificial intelligence and machine learning have raised the level of sudden fall detection [2]. They have enhanced the accuracy of detecting and tracing human poses in video footage, which can improve sudden fall detection. Some machine learning models can extract the locations of human body parts in the form of key points. These key points refer to specific points on the human body [3], such as the body, face, hands, and feet. The study of key points [4], especially in the context of body movement dynamics, becomes a fascinating aspect of this technology. These key points, acting as beacons of human body parts, provide intricate patterns according to human body movement [5]. This is particularly relevant when observing falls, which involve sudden and often unexpected changes in these patterns. Machine learning models, which have already revolutionized various sectors, offer significant promise in analyzing these key point dynamics relating to sudden falls. Although advanced machine learning models bring depth and sophistication, they also require a meticulous choice of architecture and approach. Among the numerous architectures available, transformers [3] stand out for their high accuracy, especially with their powerful attention mechanisms.

Attention mechanisms enable models to focus on certain parts of the input more than others, discerning the salient features and relationships. This is especially crucial when analyzing key points [6], where the inter-relationship and speed between certain key points can be the determining factor in detecting sudden falls. For example, an unexpected acceleration or deceleration between the nose and the center of the body could indicate a fall in progress. Incorporating the attention mechanism’s focus on the velocities of these key points can provide an unprecedented level of accuracy in detecting body motion. Each key point includes its own set of information, and when analyzed collectively, it can provide a comprehensive picture of human movement. A transformer, with its multiple layers of attention, can sift through this data, highlighting the anomalous pattern characteristic of a sudden fall. Moreover, the versatility of the transformer model makes it a suitable candidate for this endeavor. Although it was initially designed for tasks in language processing [7], its adaptability has been proven across various domains, from image recognition to time-series data prediction [8]. In the context of fall detection, its ability to parse through sequential data [9] and understand the inherent patterns and anomalies makes it a promising tool. In the proposed methodology in this study, the velocities of key points as well as their locations are integrated into the transformer’s attention mechanism. This was intended not only to enhance the reliability of fall detection but also to reduce the instances of false alarms, which has been a persistent challenge in earlier models. By emphasizing the dynamics of human body movement and leveraging the power of advanced machine learning architectures, the goal of supporting elderly people and rehabilitating patients [10] can be reached, resulting in a new era of safety, assurance, and precision in fall detection.

## 2. Literature Review

In recent years, the application of machine learning in monitoring human activities and ensuring safety has garnered significant attention from industry and academics. The development of fall detection systems using various feature extraction [11] and classification strategies [12] has been the focus of several studies. These studies can be categorized into two main types: camera-based systems [13] and wearable sensor-based systems [14]. However, there are only a few examples of sensor-based systems [15] for individual monitoring. Many studies utilize sensors such as accelerometers [16] and electromyography sensors (EMGs) [17], and they record different signals in order to collect data.

### 2.1. Camera-Based Related Systems

Camera-based systems for fall detection collect video data using cameras [18] so as to identify whether a fall has occurred. These systems gather information such as body posture, movement patterns, and the movement of critical parts of the human skeleton. Researchers such as Menacho et al. [19] utilized a camera-based system that combines dense optical flow (OF) for global characterization with feature maps within convolutional neural networks (CNNs) for local characterization. This approach used red, green, and blue (RGB) signals as input and achieved an accuracy of 88.55% with the UR (University of Rzeszow) Fall Detection dataset. Xu et al. [20] employed RGB signals and combined OpenPose with CNNs to characterize human key points for fall detection. Their approach was tested on three datasets, including the UR Fall Detection dataset [21], the Multi-cam Fall Detection Dataset [22], and the NTU RGB + D Dataset [23], achieving an accuracy rate of 91.7%. Thummala et al. [24] harnessed RGB signals as input and integrated foreground extraction via background subtraction, leveraging Gaussian Mixture Models (GMMs) for global characterization in fall detection. Applying their strategy to the LE2I dataset [25], they registered an accuracy of 95.16%. Zhang et al. [26] employed RGB signals as input and utilized CNNs for the identification of key points, specifically through convolutional pose machines and human body vector construction for local characterization. They incorporated a logistic regression classifier and considered both rotation energy sequences and generalized force sequences. While their method was evaluated on a proprietary video dataset, it achieved a fall detection rate of 98.7% and a low false-alarm rate of 1.05%.

### 2.2. Wearable Sensor-Based Systems

Wearable sensors, such as accelerometers, pressure sensors, gyroscopes, and magnetometers, are prevalently used in fall detection. Typically, the effectiveness of these wearable systems relies on specific datasets. Wearable sensors offer the benefit of being resilient across various environments and can directly record human activity data. However, they also come with a set of disadvantages, such as being bulky and invasive to wear continuously, particularly for the elderly. The obligation to wear these sensors throughout the day poses an inconvenience, compounded by limited battery life. Moreover, the precise placement of sensors plays a crucial role in the accuracy of fall detection and classification, which presents a notable challenge in the use of wearable sensors.

Wang et al. [27] incorporated an attention mechanism into the traditional CNN model to analyze sensor data features more effectively. They employed the renowned UCI-HAR dataset to gauge the effectiveness of their enhanced CNN-based HAR model that achieved an accuracy of 90.18% on a weakly labeled dataset. Tufek et al. [28] explored the proficiency of LSTM and CNN models in the classification of human activities using sensor data. Utilizing the UCI-HAR dataset for evaluation, their study discovered that the three-layer LSTM model surpassed the CNN model with 97.4% classification accuracy. Pernini et al. [29] developed a fall detection system based on various phases such as start, impact, posture, and aftermath. The system is designed to trigger alarms for critical falls where recovery is not possible. The distinction between actual falls and other activities is achieved by setting specific thresholds for acceleration and orientation, which are determined by Support Vector Machines (SVMs). Santos et al. [30] conducted a study utilizing a cloud technology platform based on the Internet of Things (IoT) in combination with a convolutional neural network, referred to as CNN-3B3 Conv, for fall detection. Rather than employing images for analysis, this study used sensors acting as accelerometers for end-users. These sensors were interconnected with a smartphone and a wristwatch affixed to the individual’s body. The model achieved an accuracy of 99.68% after data augmentation when using a smartwatch dataset. Chen et al. [31] developed a CNN model specifically tailored for accelerometer data, consisting of three convolution and three pooling layers. Using a dataset of 31,688 samples covering eight distinct activities, including falling, running, jumping, and various modes of walking, this study utilized an accelerometer sensor embedded in an Android smartphone for data collection. Compared to SVM and Deep Belief Network (DBN) methods, the CNN model outperformed them and achieved an accuracy of 93.8%.

In sensor-based systems, the prominent trend is the use of smartphone-based sensors for data collection and the application of machine learning for data analysis [32]. Additionally, researchers have explored various fall detection methods, encompassing human activity levels [33], shape characteristics (such as width-to-height ratio) [34], and motion patterns [35]. While extensive reviews have covered vision-based systems, there is a notable gap in the literature regarding fall detection systems that rely on non-vision sensors, including wearables and ambient sensors. Chen et al. [14] highlighted the significance of individual depth cameras and inertial sensors in vision- and non-vision-based systems, respectively. They emphasized that a fusion of both types of sensors can result in more robust fall detection systems compared to those relying on a single sensor modality. Xu et al. [36] focused on vision-based systems but overlooked non-vision sensors like wearables and ambient sensors. Nyan et al. [37] explored syncopes by employing a combination of a three-axis accelerometer and a two-axis gyroscope, which were attached to both the thigh and waist. Their approach involved utilizing distinctive body kinematic features to identify falls during the descending phase of body motion. With this system, Nyan achieved 100% sensitivity. However, 16% of Activities of Daily Living (ADLs) were undetected as falls, such as when shopping or using the toilet. Dai et al. [38] introduced a novel approach involving the use of mobile phones. This approach relied on a 3D accelerometer within a mobile phone to develop a fall detection algorithm. The algorithm calculated the resultant acceleration vector and vertical acceleration magnitudes, using predefined thresholds for fall detection. However, the limitation of threshold-based methods, in general, is a challenge in adapting to different individuals. Several of the methods discussed above have not been validated for real-time applications and for outside controlled laboratory environments. Furthermore, the rapid advancement of electronics has led to the availability of smaller and more cost-effective components. For example, accelerometers and cameras integrated into smartphones have emerged as practical technological choices for conducting fall detection research, as noted by Igual et al. [39].

This study introduces an application of a transformer-based model for fall detection, which significantly improves upon previous approaches that utilized traditional machine learning models like CNNs or LSTMs. By leveraging the self-attention mechanism, the transformer model captures complex dependencies in sequential data, thereby enhancing the accuracy of fall detection. Unlike earlier methods that often relied on wearable sensors or simpler key point analysis, this research utilizes key points extracted by MediaPipe, specifically targeting the body’s central point, nose, and knees to capture critical movement changes. By applying the Kalman Filter, key point data can be smoothed so as to reduce noise, resulting in more accurate velocity calculations. This approach also notably reduces false positives compared to traditional systems by effectively distinguishing falls from other movements, making it suitable for real-time applications in elderly care facilities.

## 3. Proposed Methodology

The proposed methodology in this study focuses on developing a transformer-based neural network model [40] for classifying time-series or sequential data. This advanced approach leverages the self-attention mechanism [41] intrinsic to the transformer, making it highly effective in understanding complex dependencies in sequential input data. The model architecture is designed to capture subtle patterns within the data, providing a nuanced understanding for accurate classification. Given the complexity and variability of time-series data, this methodology promises enhanced performance compared to traditional approaches, particularly in tasks where long-term dependencies or intricate patterns play a significant role.

In the context of real human pose data, this model can significantly contribute to fields such as motion analysis, activity recognition, and other areas where understanding sequential patterns is vital. Its capability to discern intricate relationships within sequential data makes it particularly suited for tasks requiring nuanced data interpretation. Looking ahead, this methodology has the potential for further enhancements, such as integrating more complex attention mechanisms or exploring different regularization techniques, to refine its predictive accuracy and utility across an even broader range of applications. Vision-based fall detection techniques often raise privacy concerns, particularly when deployed in private living spaces such as bathrooms or bedrooms. This study minimizes privacy risks by relying solely on human pose key points extracted through the MediaPipe framework, which abstracts human motion without capturing full visual information. Using only key points reduces identifiable details, making it a less intrusive method for fall detection.

### 3.1. Data Collection and Pre-Processing

Due to the challenge of finding existing datasets on fall incidents, this study creates its own experimental dataset. To ensure the data collection environment closely resembles real-life conditions, an indoor scenario similar to a CCTV surveillance setting was designed. In this experimental environment, the camera position and angle were fixed and maintained while recording the video.

The experiment was conducted by seven participants (four males and three females), providing a diversity of fall patterns across genders. Participants were recorded performing different postures (refer to Table 1), with each posture captured at roughly 10 s intervals. For the safety of participants during falls, a mattress was laid on the floor, and the participants fell onto the mattress. By installing the mattress, participants could perform posture more realistically while minimizing injury risk.

Table 1 summarizes the types of postures and their corresponding data counts. A total of 600 video clips was collected, with 100 samples per posture. To capture a diverse range of scenarios and environmental conditions, data collection was spread over several days rather than performing all postures at once.

In the initial phase of data preprocessing, where the collected video clips exhibited variability in length and frame numbers, a standardization of frame numbers was adopted to ensure data length uniformity, which is crucial in transformer models [42]. Among all recorded video clips, a video clip with the fewest frames was identified, and the frame number of this video clip was set to as standard frame number. This benchmark was important for randomized sampling in subsequent data extraction, ensuring dataset diversity [43]. Thereafter, for each video clip, an equal number of frames with the standard frame number was randomly chosen. This process was crucial for data balance and variety in enhancing model training. The selected frames were carefully arranged to ensure that the structure of the data was consistent, even though the original video clips varied in length and frame count. This means that after the frames were selected, they were placed in the correct order to preserve the sequence of actions in each video.

Aligning the length of video clips in this manner ensured that the model would be trained on a consistent and balanced set of data, enhancing the reliability and validity of the training outcomes. This meticulous standardization process not only addresses variability but also maximizes the dataset’s utility in developing the model.

Finally, each video frame, including positional and characteristic information (e.g., coordinates, visibility), was extracted into a new dataset for model development. This structured dataset became the foundation for further analysis and the training of machine learning models.

### 3.2. Selection of Key Points and Calculation of Key Point Change Speed

MediaPipe [44] is a highly versatile framework developed by Google to build pipe-lines for processing perceptual data, such as video, audio, and sensor streams. When it is applied to human pose data, it offers a state-of-the-art solution for extracting key points [3], specifically through its Pose module. MediaPipe Pose is designed to detect and track the human body through key points in real time, even in complex environments. It employs machine learning models that process images and video streams to identify and locate key points of the human body, such as the elbows, knees, shoulders, and hips. The model is efficient and lightweight, which makes it suitable for real-time applications on both CPU and mobile devices [44].

**Figure 1:**
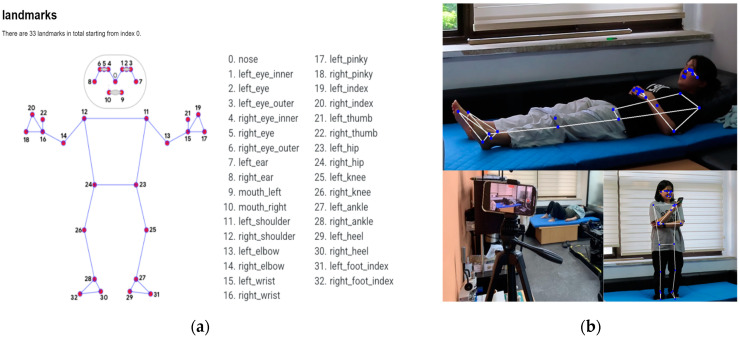
(**a**) MediaPipe skeletal framework [3]; (**b**) Experimental environment and key points by MediaPipe Pose.

**Figure 2:**
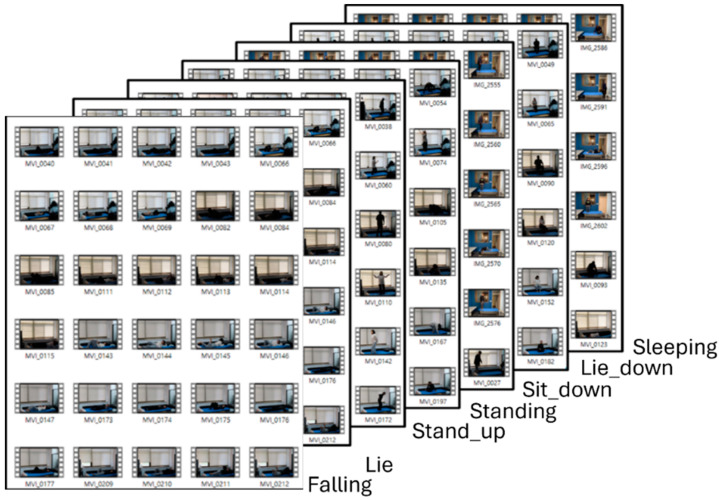
Video clips for MediaPipe Pose on each posture class.

MediaPipe Pose takes image or video frames, represented in, as input data. The model processes a frame to identify the region of interest that is typically where the human figure is located. The output is a set of coordinates (*x*, *y*, and *z*) for each detected key point. Notably, 33 distinct key points are extracted as landmarks [45] (refer to Figure 1a and the images in Figure 1b). These coordinates can be used to understand posture, track movements, or serve as input data for further analysis such as activity recognition or biomechanical studies. MediaPipe Pose stands out for its ease of use, flexibility, and efficiency, so it is regarded as a go-to solution for developers and researchers work-ing with pose estimation and human movement analysis. Figure 2 shows video data with postures).

In this study, key points from both shoulders (11 and 12 in Figure 1a) and both hips (23 and 24 in Figure 1a) are used to generate the center of body named ‘central_point’. The purpose of defining ‘central_point’ is to trace the movement speed of the human body in detecting a sudden fall. The calculation of ‘central_point’ is performed by the following method: First, the midpoint ($M_{1}$) between the left shoulder ($p_{1}$) and the right hip ($p_{2}$) is calculated:

(1) $$M_{1}=(\frac{p_{1_{x}}+p_{2_{x}}}{2},\;\frac{p_{1_{y}}+p_{2_{y}}}{2},\;\frac{p_{1_{z}}+p_{2_{z}}}{2})$$

The subscripts x, y, and z represent the three-dimensional coordinates of p and are extracted from MediaPipe Pose. Then, the midpoint ($M_{2}$) between the right shoulder ($p_{3}$) and the left hip ($p_{4}$) is calculated:

(2) $$M_{2}=(\frac{p_{3_{x}}+p_{4_{x}}}{2},\;\frac{p_{3_{y}}+p_{4_{y}}}{2},\;\frac{p_{3_{z}}+p_{4_{z}}}{2})$$

Then, the ‘central_point’, C, is calculated as the midpoint between two right and left midpoints ($M_{1},\;M_{2}$).

(3) $$C=\left(\frac{M_{1_{x}}+M_{2_{x}}}{2},\;\frac{M_{1_{y}}+M_{2_{y}}}{2},\;\frac{M_{1_{z}}+M_{2_{z}}}{2}\right)$$

C is used to track the movement speed and provides information on how quickly the upper body is moved. It provides a representative measure of the upper body’s overall motion and is particularly helpful when discriminating balance and fall likelihood. Moreover, after applying the Kalman Filter [12] to smooth the 3D coordinate data of the human key points, the velocity of each key point is calculated. The Kalman Filter is an algorithm designed to estimate the state of a system based on observed data and allows for more accurate predictions by reducing the impact of noise. In this study, a Kalman Filter is employed to reduce noise in the coordinates of key points and to enhance the robustness and accuracy of velocity of key points. This filter combines previous states with current observations to compute the optimal estimates. To account for potential p in the key point data, a Kalman Filter was applied to smooth the extracted key point coordinates. This filtering approach helps estimate key point positions even during temporary occlusions, providing more reliable midpoint calculations. The application of Kalman Filter is computed as follows:

(4) $${\hat{x}}_{k}=A{\hat{x}}_{k-1}+Bu_{k}+K(z_{k}-H{\hat{x}}_{k-1})$$

Here, ${\hat{x}}_{k}$ represents the state variable. $u_{k}$ denotes the control input. $z_{k}$ is the observed value. A is the state transition matrix and B is the control matrix. H indicates the observation matrix and K is the Kalman Gain.

Using the smoothed 3D coordinate data after applying the Kalman Filter, the velocity of at each time step is calculated. Velocity is defined as the Euclidean distance between two consecutive points in time and is computed as follows:

(5) $$u_{t}=\left\|P_{t}-P_{t-1}\right\|$$

Here, $u_{t}$ represents the unit velocity at time t, and $P_{t}$ denotes the position at time t. The velocity is initialized as 0, which assumes no movement at the initial state.

Using this calculation, the velocities of the ‘central_point’ as well as the head, left knee, and right knee are calculated. This velocity adds more information for detecting sudden fall so that location and movement are considered concurrently, which improves the performance of sudden fall detection. These four key points (central_point, left knee, and right knee) are chosen as they are expected to exhibit the most significant differences in movement patterns between daily activities and sudden falls for humans.

## 4. Experiments and Results

### 4.1. Definition of Normalization, Input Data and Output Data

The input layer of a transformer model is designed to handle sequential data, typically requiring data to be in a uniform format. Each input sequence is processed as a set of embeddings, which represent the data points in a multi-dimensional space. Transformers do not inherently preserve the order of input data; therefore, positional encodings are added to the input embeddings to maintain the sequence information. This allows the model to understand the relative positioning of each data point within the sequence, which is crucial for tasks like fall detection where the order of movement of key points matters. The input layer is the initial point of data entry, which refers to the process of feeding the model with standardized input data for fall detection. The input data consist of the normalized coordinates of key points extracted by MediaPipe, including the velocities of selected key points like the body’s central point, nose, and knees. This data entry is essential for the model to interpret sequential human body movements and detect falls based on the time-series data input. For this study, the input data consist of the coordinates of specific key points extracted using MediaPipe, such as the central point of the body, nose, left knee, and right knee. These key points are chosen as they are expected to exhibit the most significant movement changes of the human body during sudden falls. The data also include the instantaneous speeds of these key points, which are calculated to capture the dynamics of movement.

The key point coordinates are first smoothed using a Kalman Filter to reduce noise among video frames to ensure more reliable speed calculations. The resulting data are then normalized using Min-Max scaling to fit within a consistent range, which makes it suitable for model training. This normalized dataset is fed into the transformer as a series of time-ordered frames, and each represents the spatial and velocity data of key points at a given moment.

The output of the transformer model is a classification of the detected activity as a posture type (Table 2), specifically identifying whether the movement sequence corresponds to a fall or other actions (e.g., standing, sitting, lying down). The final output layer of the transformer uses a SoftMax activation function to assign probabilities to each possible action, with the highest probability indicating the detected posture. This setup allows the model to discern between different movement patterns and accurately classify sudden falls. Table 2 shows a structure and a sample from the original data collected.

Data normalization [46] is performed to adjust each data point as a uniform range, which helps the model effectively learn from data with varying scales. This approach maintains consistent data distribution and prevents the learning process from being distorted by extreme values. To standardize the data, Min-Max normalization is employed so that all coordinate data are converted to values ranging between 0 and 1. Min-Max normalization is defined as follows:

(6) $$x_{norm}=\frac{x-x_{min}}{x_{max}-x_{min}}$$

where x represents the original data value. $x_{min}$ is the minimum value in the dataset, and $x_{max}$ is the maximum value in the dataset. $x_{norm}$ is the normalized data value.

Normalization is performed on the key points data obtained from the MediaPipe Pose pipeline, which is essential for preparing the data for a transformer model [47]. This process involves scaling the 33 landmarks, ensuring they fall within a consistent range, typically between 0 and 1. This is critical for the transformer model’s performance, as it relies on standardized input for accurate pattern recognition and learning. By normalizing the data, disparities caused by different scales or variations in the key points can be minimized, which leads to more reliable and effective model training and analysis. Normalized data are represented in Table 3.

### 4.2. Transformer Encoder-Decoder Architecture with Attention Mechanism

The core of the transformer model is the encoder layers [48]. Each encoder consists of two main components: a multi-head self-attention mechanism [49] and a position-wise fully connected feed-forward network [50]. The multi-head self-attention mechanism allows the model to weigh the importance of different parts of the input sequence differently, providing a dynamic aggregation of information. This is crucial for understanding the context of each posture within a sequence. Transformers do not inherently process sequential data as sequential; they treat inputs as sets. To account for the order of data, positional encodings are added to the input embeddings. These encodings give the model information about the position of each item (key point) in the sequence. This way, the model can distinguish between sequences with the same items and key points in different orders, which is vital for interpreting time-series data correctly. After passing through several transformer encoder layers, the output of the mode is a sequence of vectors representing the transformed input sequence. The final layer of the transformer model is typically a fully connected layer that maps these vectors to the desired output shape, which is a multi-class classification problem: falling, lie, lie down, sit down, sleeping, standing, and stand up. The activation function for this layer used in transformer is a SoftMax since it is a multi-class classification task.

The attention mechanism in deep learning imitates cognitive attention [41] by allowing models to focus on specific parts of the input sequentially, rather than processing the entire dataset at once. This is particularly beneficial for sequential data where the relevance of a specific data point might depend on others in the sequence. The encoder processes the input sequence (a series of key points from pose data) and converts it into a context-rich representation. In the pose data, this would involve capturing the spatial relationships and dependencies between different key points at each frame in sequence.

The decoder [51] then generates the output sequence as one element at a time. With an attention mechanism, the decoder can “attend” to different parts of the encoder’s output at each step. For instance, in pose-based activity recognition, the decoder might focus on the movement of arms in one step and the legs in another, depending on the activity being performed. The model calculates attention weights [52], signifying the importance or relevance of each part of the encoder’s output to the current step of the decoder. These weights are then used to create a weighted combination of the encoder outputs, forming a context vector. This context vector is tailored to the decoder’s current step, ensuring that the model focuses on the most relevant information from the input sequence.

**Figure 3:**
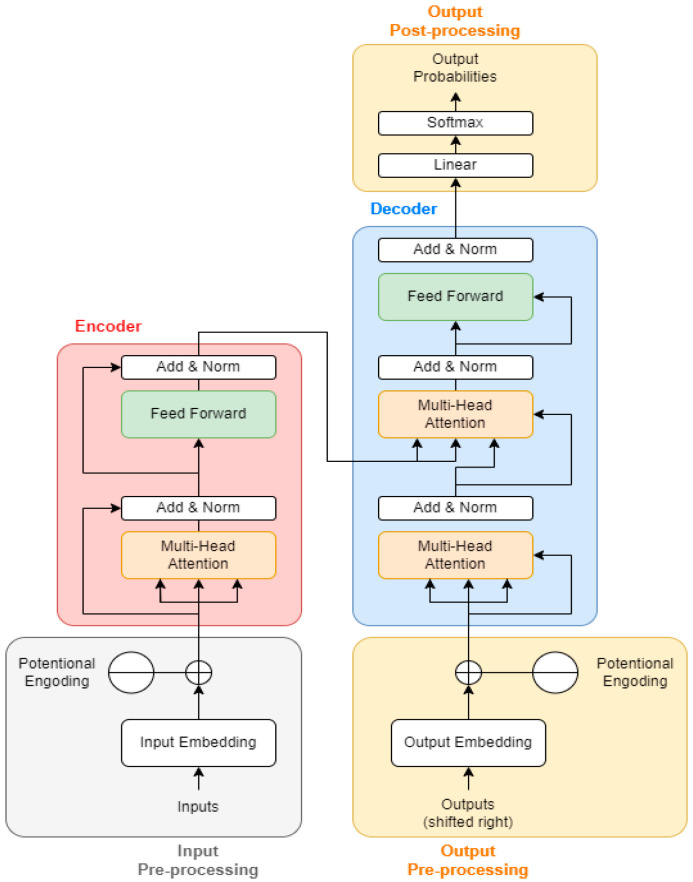
Transformer model structure [54].

When analyzing pose data, the attention mechanism allows the model to focus on specific key points that are more relevant at different stages of an activity. For example, in swimming, the model might focus more on the arms’ position during the initial stages and more on the legs during the latter stages. The attention mechanism leads to better performance in tasks like sequence-to-sequence prediction, translation, or time-series analysis, as it enables the model to handle long-range dependencies and capture nuanced relationships in the data [53]. It offers the better interpretability of the model behavior, as one can analyze which parts of the input sequence the model is focusing on at each step. Figure 3 describes the structure of the transformer model.

### 4.3. Model Architecture and Training

Transformer-based neural network architecture is mainly used for sequential data analysis. The model begins with an input layer tailored to the preprocessed data’s dimensions. It then progresses through several transformer blocks, each composed of layer normalization, multi-head attention mechanisms (to capture diverse data aspects), dropout (for regularization), and residual connections (facilitating gradient flow during backpropagation). Following the transformer blocks, global average pooling reduces the data dimensionality, succeeded by a Multi-Layer Perceptron (MLP) [55] with dense layers featuring ReLU activation and dropout [56]. The final layer, a dense SoftMax activation layer, classifies the input into predefined categories. Training involves the Adam optimizer with a learning rate that adjusts over time (Cosine Decay), and to mitigate overfitting, early stopping is strategically employed based on validation loss according to the proposed method.

The model’s efficacy is rigorously evaluated using the separated test dataset. Performance metrics such as accuracy and loss provide insights into the model’s classification capabilities. To enhance generalizability and curb overfitting, L2 regularization is integrated into the dense layers, imposing penalties on large weights to encourage the learning of more general patterns rather than overfitting to the training data. Then, the model’s detailed evaluation through a multi-classification report offers in-depth insights into its precision, recall, and F1-score across different classes, affirming its applicability and robustness in classifying falling, lie, lie down, sit down, sleeping, standing, and standup classes.

Moreover, various neural network configurations and training parameters were employed to evaluate the model’s performance under different conditions. Specifically, multiple architectures (LSTM [57], GRU [58], and transformer) and optimization methods (Adam, SGD, and AdamW) were tested to explore the robustness of the model. Each configuration provided insight into the model’s sensitivity to different hyperparameters and helped identify the optimal setup for fall detection tasks.

The selection of different optimization methods broadened the scope of analysis. For instance, AdamW [59] was chosen for its ability to handle sparse gradients, which is advantageous when working with complex time-series data, while stochastic gradient descent (SGD) [60] offered perspectives on model performance with traditional gradient descent techniques. This approach informed the selection of the final model configuration, which achieved a balance of accuracy and efficiency.

Although using multiple configurations introduced variability, it also yielded valuable insights into the model’s robustness and performance sensitivity. This systematic exploration underscores the effectiveness of the transformer model with attention mechanisms in fall detection applications.

## 5. Results and Analysis

### 5.1. Performance Measure

In this study, precision and the F1-score are chosen as critical metrics to evaluate the performance of the transformer-based fall detection model. These metrics provide insight into the accuracy and reliability of AI model, which are crucial for real-world applications like monitoring human activity.

Precision measures the proportion of correctly identified fall events among all instances that the model predicted as falls. It is calculated using the following formula:

(7) $$Precision=\frac{TP}{TP+FP}$$

where TP (true positives) represents the correctly predicted falls and FP (false positives) indicates non-fall events that are incorrectly predicted as falls. A high precision value suggests that the model is effective at minimizing false alarms, which is important to avoid unnecessary interventions and reduce the burden on caregivers or monitoring systems. The F1-score provides a balanced measure of the model performance by combining precision and recall. Recall is the proportion of actual fall events that are correctly identified by the model and calculated as

(8) $$Recall=\frac{TP}{TP+FN}$$

where FN (false negatives) are actual falls that the model failed to detect. The F1-score is the harmonic mean of precision and recall, and is given by

(9) $$F1-score=2\times \frac{Precision\times Recall}{Precision+Recall}$$

The F1-score is particularly valuable in fall detection because it accounts for both false positives and false negatives, offering a comprehensive view of the model performance. A high F1-score indicates that the model not only accurately identifies falls but also does so consistently, balancing the need to detect true falls while avoiding missed detections.

Using these metrics allows for a robust evaluation of the effectiveness of model in distinguishing falls from other activities, which is essential in reducing both the number of missed falls and false alerts. This comprehensive assessment helps ensure that the model can be reliably deployed in practical environments, providing accurate and timely fall detection that enhances safety for elderly individuals.

### 5.2. Experimental Results

Table 4 provides a comparison of the three models—LSTM [61], GRU [62] and transformer—used for fall detection based on key points extracted by MediaPipe and normalized data.

According to Table 4, various LSTM, GRU, and transformer models have been configured with distinct hyperparameters. LSTM1, utilizing the Adam optimizer [63], with a learning rate of 0.01, 64 units, a dropout rate of 0.2, and a batch size of 32, shows an F1-score of 0.922 and a precision of 0.927. LSTM3, employing AdamW [64], with an identical learning rate and units but a higher dropout rate of 0.4 and a larger batch size of 64, yields the highest accuracy among LSTM models, with an F1-score of 0.955 and a precision of 0.958. Similarly, GRU3, with AdamW, 128 units, and a 0.4 dropout rate, achieves an F1-score and a precision of 0.873 and 0.903, respectively. Among transformer models, Transformer 3, adopting AdamW, a higher dropout rate of 0.4, and a batch size of 64, achieves an F1-score of 0.974, with a precision score of 0.976, indicating superior performance with these settings. Notably, the models incorporate key layers such as Layer Normalization, Multi-Head Attention, Conv1D, GlobalAveragePooling1D, and Dense, as denoted by t1 to t5.

**Figure 4:**
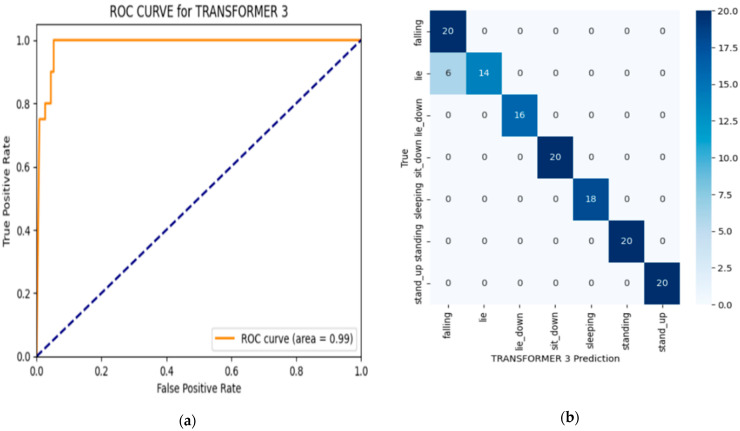
(**a**) ROC; (**b**) confusion matrix.

Transformer 2, which uses the Adam optimizer but with a higher dropout rate of 0.3 compared to Transformer 1, performs exceptionally well, with an accuracy of F1-score of 0.964 and a precision score of 0.962. These results are very close to those of Transformer 3, indicating that both configurations yielded strong performance in fall detection. However, Transformer 2 has a slightly lower accuracy compared to Transformer 3, which is the best-performing model according to Table 4. To further validate the prediction performance of the predictive model with the highest performance, a binary classification technique was used to measure classification performance through a fall-detection-class confusion matrix [65]. We also present an ROC (Receiver Operating Characteristic) curve [66] in Figure 4 for the transformer.

The ROC (Receiver Operating Characteristic) curve evaluates the model’s performance by plotting the true-positive rate (sensitivity) against the false-positive rate across different classification thresholds. The area under the ROC curve (AUC) provides an overall measure of the model’s ability to distinguish between classes. In this case, the AUC of 0.99 indicates the model’s effectiveness in differentiating between fall and non-fall events, with high sensitivity and a low false-positive rate, as represented in Figure 4a. The confusion matrix provides a detailed view of the classification results for the transformer model. Each row represents the actual classes (true labels), while each column represents the predicted classes. The diagonal elements indicate the correct predictions for each class, such as the 20 correctly predicted instances of the “falling” class. Off-diagonal elements represent misclassifications—for example, the six instances where the model predicted “lie” instead of “falling”. This confusion matrix is useful for assessing the model’s accuracy by illustrating both correct classifications (true positives) and misclassifications (false positives and false negatives). It enables the calculation of key performance metrics, such as the precision, recall, and F1-score for each posture class. High values along the diagonal indicate accurate predictions, with minimal values in the off-diagonal cells reflecting fewer misclassifications, as represented in Figure 4b. Together, the ROC curve and confusion matrix help to demonstrate the model’s ability to classify posture types accurately, which is important for fall detection.

The evaluation of the proposed algorithm was conducted on an acquired dataset, with results compared to other studies in the field to contextualize its performance. This comparison demonstrates that the transformer-based model, leveraging attention mechanisms, offers improvements over conventional fall detection methods, particularly in terms of accuracy and the reduction of false positives. Additionally, an analysis of correctly and incorrectly classified instances was performed to provide further insights into the model’s reliability. This analysis revealed that misclassifications were predominantly due to subtle, complex movements that closely resemble fall patterns, highlighting areas for further refinement. Such an approach underscores the model’s contributions beyond dataset-specific performance, validating its applicability across a broader range of real-world scenarios.

## 6. Conclusions and Future Work

This study proposes a novel machine learning model utilizing a transformer architecture to predict sudden fall incidents by analyzing key points and movements derived from the human body. The primary focus of this paper is applying a new machine learning model for health monitoring, and it provides practical applications for elderly care facilities, where timely sudden fall detection is crucial. The model used analyzes video data using MediaPipe to track continuous positions of key points and calculate the instantaneous speed of key points. The unique aspect of an attention mechanism within the transformer model results in the effective recognition of critical patterns of position and speed shifts indicating sudden falls. The proposed method can be characterized by addressing the challenge of precise and timely fall detection by focusing on the dynamics of human body movement rather than static poses. Experimental tests on datasets established by this research demonstrated that this approach significantly enhances sudden fall detection accuracy and notably reduces false positives, which is a common issue in existing fall detection systems. By concentrating on the speed changes of key points, the proposed method offers a comprehensive understanding of human body movement, and it can improve the identification of potential fall events.

This study tests various machine learning models, including conventional models, for comparative analysis. The transformer-based approach shows superior performance in detecting sudden falls, as evidenced by its high accuracy and the low rate of false positives. However, this study acknowledges certain limitations such as the dependency on the quality and variability of video data and challenges in real-time data processing. In future work, the authors will explore enhancements to the transformer model, including more complex attention mechanisms and different regularization techniques, to refine its predictive accuracy. 

Additionally, integrating this model with real-time monitoring systems in healthcare and elderly care can vastly improve the timely detection and prevention of falls, which could potentially save lives and reduce healthcare costs. The next steps also involve adapting the proposed method for various environments and conditions, ensuring its effectiveness across diverse scenarios. However, a limitation of this study is the use of self-induced falls, which may not fully reflect the characteristics of accidental falls in older adults. Additionally, the study involved participants under sixty-five, which may differ from the target demographic of elderly populations. Future studies should aim to include participants aged sixty-five and older to increase the ecological validity of the results.

## Figures and Tables

**Table 1:** Posture types and corresponding data descriptions.

Posture Type	Number of Video Clip	Posture Description
Falling	100	Falling posture
Stand_up	100	Standing posture
Standing	100	Transitioning from lying down or sitting to standing
Sit_down	100	Sitting posture
Lie_down	100	Lying down posture
Sleeping	100	Sleeping posture with less movements
Lie	100	Transitioning from standing to lying down

**Table 2:** Original data sample from MediaPipe.

Index	Frame	0_x_ (Nose)	0_y_ (Nose)	0_z_ (Nose)	1_x_ (Left_Eye_Inner)	1_y_ (Left_Eye_Inner)	…	Central_Body_Speed	Left_Knee_Speed	Right_Knee_Speed	Y
1	1	0.379765	0.553287	0.208805	0.381411	0.55061	⋮	0	0	0	falling
	2	0.362753	0.185408	−0.32835	0.364276	0.17145	⋮	0.11449	0.05578	0.083724	
	3	0.362748	0.185095	−0.30977	0.364278	0.171292		0.033503	0.020768	0.015855	
	⋮	⋮	⋮	⋮	⋮	⋮	⋮	⋮	⋮	⋮	
	9899	0.249462	0.940241	−0.25696	0.239478	0.939346		0.001084	0.001716	0.006252	
	9900	0.253466	0.935217	−0.20723	0.242784	0.934585	⋮	0.003875	0.003117	0.000977	
100	9901	0.251388	0.93537	−0.21042	0.243521	0.931843	⋮	0.001172	0.001893	0.002287	
⋮	⋮	⋮	⋮	⋮	⋮	⋮	⋮	⋮	⋮	⋮	
1	1	0.494136	0.256956	−0.41847	0.497415	0.239821	⋮	0.002505	0.018862	0.013497	standing
	2	0.490758	0.246923	−0.40555	0.493804	0.230732	⋮	0.004472	0.003269	0.002368	
	3	0.488241	0.242574	−0.40686	0.490833	0.227228	⋮	0.007852	0.009989	0.005957	
	⋮	⋮	⋮	⋮	⋮	⋮	⋮	⋮	⋮	⋮	
	9899	0.507184	0.241033	−0.35604	0.512011	0.230799		0.005119	0.031786	0.006606	
	9900	0.505589	0.240715	−0.34864	0.510574	0.230771	⋮	0.002204	0.013514	0.006841	
100	9901	0.496076	0.237336	−0.33496	0.501637	0.227336	⋮	0.001037	0.006635	0.004	

**Table 3:** Normalized data.

Index	Frame	0_x_ (Nose)	0_y_ (Nose)	0_z_ (Nose)	1_x_ (Left_Eye_Inner)	1_y_ (Left_Eye_Inner)	…	Central_Body_Speed	Left_Knee_Speed	Right_Knee_Speed	Y
1	1	0.374411	0.569179	0.685071	0.372001	0.577581	⋮	0	0	0	falling
	2	0.353146	0.244493	0.346481	0.350831	0.244334	⋮	0.280930	0.180228	0.233038	
	3	0.353140	0.244216	0.358191	0.350833	0.244196		0.082207	0.067104	0.044132	
	⋮	⋮	⋮	⋮	⋮	⋮	⋮	⋮	⋮	⋮	
	9899	0.211527	0.910703	0.391483	0.196643	0.919246		0.002661	0.005545	0.017402	
	9900	0.216533	0.906268	0.422828	0.200727	0.915062	⋮	0.009507	0.01007	0.002718	
100	9901	0.213935	0.906404	0.42082	0.201638	0.912651	⋮	0.002876	0.006116	0.006367	
⋮	⋮	⋮	⋮	⋮	⋮	⋮	⋮	⋮	⋮	⋮	
1	1	0.517380	0.30764	0.289674	0.515324	0.304426	⋮	0	0	0	standing
	2	0.513157	0.298785	0.297817	0.510864	0.296438	⋮	0.006145	0.060945	0.037568	
	3	0.510012	0.294947	0.296995	0.507193	0.293358	⋮	0.010973	0.010563	0.006591	
	⋮	⋮	⋮	⋮	⋮	⋮	⋮	⋮	⋮	⋮	
	9899	0.533691	0.293586	0.329027	0.533359	0.296497		0.012561	0.102704	0.018387	
	9900	0.531697	0.293306	0.33369	0.531582	0.296472		0.005409	0.043665	0.019042	
100	9901	0.519806	0.290323	0.342313	0.520541	0.293453	⋮	0.002543	0.021437	0.011134	

**Table 4:** Prediction results.

Model	Model Parameters					Results	
LSTM	optimizers	Learning rate	Unit	Drop out. rate	Batch size	Performance	
						F1-score	Precision
LSTM1	Adam	0.01	64	0.2	32	0.922	0.927
LSTM2	SGD	0.01	64	0.3	32	0.916	0.921
LSTM3	AdamW	0.01	64	0.4	64	0.955	0.958
GRU1	Adam	0.01	64	0.2	32	0.946	0.949
GRU2	SGD	0.01	64	0.3	32	0.657	0.668
GRU3	AdamW	0.01	128	0.4	64	0.873	0.903
TRANSFORMER	optimizers	Layer	Drop Out. rate	Activation Function	Batch size	Performance	
						F1-score	Precision
TRANSFORMER 1	Adam	t1, t2, t3, t4, t5	0.1	Relu	32	0.918	0.921
TRANSFORMER 2	Adam	t1, t2, t3, t4, t5	0.3	Relu	32	0.964	0.962
TRANSFORMER 3	AdamW	t1, t2, t3, t4, t5	0.4	Relu	64	0.974	0.976

t1 LayerNormalization; t2 MultiHeadAttention; t3 Conv1D; t4 GlobalAveragePooling1D; t5 Dense.

## Data Availability

Dataset available on request from the authors.
